# Comparison of imaging parameters pre- and post- reductive procedure for atlantoaxial dislocation via posterior fixation using pedicle screw and rod: a cross-sectional study

**DOI:** 10.1186/s12891-019-2842-3

**Published:** 2019-10-15

**Authors:** Jia Shao, Yanzheng Gao, Kun Gao, Zhenghong Yu

**Affiliations:** Department of Spinal Surgery, Henan provincial people’s hospital, Weiwu road No 7, Jinshui district, Zhengzhou City, Henan province China

**Keywords:** Radiological measurement, Atlantoaxial dislocation, Lateral atlantoaxial interval, Lateral mass interval, Post-operative

## Abstract

**Background:**

To compare the imaging parameters pre- and post- reductive procedure for atlantoaxial dislocation via posterior fixation using pedicle screw and rod.

**Methods:**

Thirty-seven patients suffering from atlantoaxial dislocation underwent posterior reduction and internal fixation by pedicle screw and rod. We measured pre-operative and post-operative atlantodental interval (ADI), clivus-canal angle (CCA), cervicomedullary angle (CMA), sum of lateral mass interspace (SLMI) of the operation and the control group. ADI, CCA, CMA, and SLMI between the pre-operative and post-operative conditions of the operation group and the control group were compared.

**Results:**

The ADI, CCA, CMA, and SLMI in the pre-operative condition of the operation group were 8.3 ± 4.3 mm, 130.2 ± 14.2°, 133.8 ± 16.7°, and 3.7 ± 1.3 mm, respectively, those in the post-operative condition of the operation group were 1.0 ± 0.9 mm, 148.5 ± 9.4°, 156.0 ± 8.2°, and 8.0 ± 2.7 mm, respectively, while those in the control group were 1.2 ± 0.3 mm, 152.7 ± 5.3°, 160.2 ± 6.3°, and 4.5 ± 1.0 mm respectively. Post-operative ADI, CCA, CMA, and SLMI were statistically different (*p* < 0.01) from pre-operative assessments. The SLMI has no significant difference between the pre-operative condition and the control group. Post-operative SLMI was statistically different from that of the control group.

**Conclusions:**

The lateral mass joints were widened after the anatomical reduction of atlantoaxial dislocation by pedicle screw and rod. Widening of the lateral mass exists in both atlantoaxial fusion and occipital-cervical fusion.

## Background

Atlantoaxial dislocation is a rare condition caused by the loss of atlantoaxial stability leading to an abnormal anatomic relationship between the atlas and the axis [1, 2]. Atlantoaxial joint stability can be destructed by rheumatoid arthritis [3], ankylosing spondylitis [4], upper cervical spinal malformation [5], or trauma [6, 7], leading to upper cervical spinal cord compression, which presents as numbness of limbs, disability, or paralysis. Surgical treatment is recommended when the spinal cord is compressed. The traditional surgical procedures include odontoid resection, laminectomy, and decompression of the foramen magnum. These procedures cannot treat atlantoaxial dislocation and may lead to worsening of the local stability, which can cause a high recurrence rate and poor long-term effects. A comparatively more ideal procedure is reduction and internal fixation of the atlantoaxial complex to regain the correct anatomical position and alignment, and correct the instability through radical reconstruction by bone grafting and fusion.

Anatomical research and radiological examination of the atlantoaxial joint have been well described. Chamberlain [8], McGregor [9], and McRae [10] presented their own standard for assessing the atlantoaxial dislocation caused by basilar invagination. Atlantodental interval (ADI) is one of the most useful parameters to describe the severity of dislocation. Clivus-canal angle (CCA) and cervicomedullary angle (CMA) are both relatively reliable parameters to estimate the compressive severity of the upper cervical spinal cord. ADI, CCA, and CMA are frequently used to evaluate the reductive effect after surgical treatment [6, 11–13]. However, these parameters all have their limitations. We found that the lateral mass may be misaligned when the dislocation of the atlantoaxial joint has been anatomically reduced according to ADI, CCA, and CMA. The purpose of this study was to compare the radiographic parameters pre- and post- reductive procedure of atlantoaxial dislocation via posterior fixation using pedicle screw and rod, and thus to provide a reference for reduction of atlantoaxial dislocation.

## Methods

### Demographic data

We retrospectively analyzed imaging parameters of a series of patients suffering from atlantoaxial dislocation between January 2015 and June 2018. The inclusion criteria was as follows: (1) Patients diagnosed with atlantoaxial dislocation. (2) Surgically treated which was confirmed by at least 2 upper cervical spine experts based on postoperative imaging. (3) Pre- and post-operative cervical X-ray, CT and MRI imaging were available. (4) Clinical follow-up was over 12 months. The exclusion criteria was as follows: (1) Patients with partial reduction or no reduction. (2) Imaging studies unavailable or unidentifiable. (3) Partial or no reduction of atlantoaxial dislocation which was confirmed by at least 2 upper cervical spine experts based on postoperative imaging. (4) Insufficient follow-up. Of all the 45 surgically treated patients, 41 patients were anatomically reduced. Of these 41 patients, a total of 37 patients (13 men) with an average age of 47.8 ± 12.1 years (12–76 years) completed the follow-up, and was included in the study group. Mean follow-up was 24.8 ± 8.6 months (12–50 months) with a follow-up rate of 90.2% (37/41). The dislocation was caused by atlas occipitalization (17 cases), os odontoideum (7 cases), rheumatoid arthritis (3 cases), ankylosing spondylitis (1 case), odontoid fracture nonunion (1 case), some other malformation (2 cases) and unknown reasons (6 cases), for more details in Table 1. Twenty-eight volunteers (12 men) without spinal disorders were incorporated in the control group. The study was approved by the Medical Scientific Research Ethics Committee of Henan Provincial People’s Hospital. All of the 37 patients and 28 volunteers gave written informed consent for participating in this study.

**Table 1:** Demographic data in atlantoaxial dislocation cases

Patient ID	Etiology	Anterior release	Fixation
1	Unknown	N/A	C1–2
2	Os odontoideum	N/A	C1–2
3	Unknown	N/A	C1–2
4	Rheumatoid arthritis	N/A	C1–2
5	Rheumatoid arthritis	N/A	C1–2
6	Atlas occipitalization, C2–3 fusion	N/A	C0–3
7	Rheumatoid arthritis	N/A	C1–2
8	Ankylosing spondylitis	Retropharyngeal	C1–2
9	Other malformation, posterior arch defect	N/A	C1–2
10	Atlas occipitalization	N/A	C0–2
11	Unknown	N/A	C1–2
12	Atlas occipitalization, C2–3 fusion	N/A	C0–3
13	Atlas occipitalization, C2–3 fusion	N/A	C0–3
14	Atlas occipitalization, C2–3 fusion	N/A	C0–3
15	Unknown	N/A	C0–2
16	Os odentium	N/A	C1–2
17	Atlas occipitalization, C2–3 fusion	N/A	C0–3
18	Atlas occipitalization, C2–3 fusion	Transoral	C0–3
19	Atlas occipitalization	N/A	C0–2
20	Atlas occipitalization	N/A	C0–2
21	Os odontoideum	N/A	C1–2
22	Odontoid fracture nonunion	N/A	C1–2
23	Unknown	N/A	C1–2
24	Unknown	N/A	C1–2
25	Atlas occipitalization	N/A	C0–2
26	Atlas occipitalization	Retropharyngeal	C0–2
27	Atlas occipitalization	N/A	C0–2
28	Os odontoideum	N/A	C1–2
29	Os odontoideum	N/A	C1–2
30	Atlas occipitalization	N/A	C0–2
31	Atlas occipitalization, C2–3 fusion	Retropharyngeal	C0–3
32	Os odontoideum	N/A	C1–2
33	Os odontoideum	N/A	C1–2
34	Other malformation, C2–3 fusion	N/A	C1–2
35	Atlas occipitalization, C2–3 fusion	Transoral	C0–3
36	Atlas occipitalization, C2–3 fusion	N/A	C0–3
37	Atlas occipitalization, C2–3 fusion	Retropharyngeal	C0–4

**Table 2:** Pre- and post- operative imaging parameters in atlantoaxial dislocation cases

Patient ID	ADI (mm)		CCA (°)		CMA (°)		SLMI (mm)	
	Pre-op	Post-op	Pre-op	Post-op	Pre-op	Post-op	Pre-op	Post-op
1	5.62	0.97	125.84	133.59	131.34	142.80	6.58	9.83
2	3.90	0.00	122.59	147.24	135.02	153.89	5.26	7.16
3	3.20	0.78	135.57	151.11	149.33	162.23	4.93	5.43
4	5.15	0.92	138.67	162.34	144.22	164.22	2.62	6.86
5	3.72	1.07	154.22	161.21	155.53	166.81	5.74	7.66
6	5.56	1.17	113.02	146.31	130.48	151.87	3.98	9.85
7	9.05	0.60	135.40	147.93	137.34	145.49	5.63	7.06
8	10.64	1.88	133.93	149.63	131.67	162.04	2.06	8.18
9	8.91	1.74	138.53	154.58	129.85	149.27	4.99	6.59
10	16.46	2.18	159.33	165.32	152.32	162.00	2.26	9.10
11	13.01	1.53	113.90	138.11	131.43	150.55	2.97	4.45
12	9.16	0.00	134.19	160.67	138.67	164.27	3.29	9.20
13	10.28	1.73	136.40	158.15	133.74	165.33	3.79	5.80
14	8.79	1.20	112.46	138.78	109.30	155.45	2.82	7.14
15	3.55	1.01	136.38	151.00	143.92	155.95	2.94	3.42
16	2.65	0.70	122.31	146.78	141.20	153.84	4.60	8.07
17	6.71	0.00	131.59	151.10	136.97	155.65	2.26	16.13
18	20.95	3.98	94.57	134.76	87.66	134.20	4.35	9.53
19	10.67	2.39	141.89	167.47	142.13	169.67	4.92	5.49
20	5.37	1.05	126.60	145.90	135.51	158.43	4.63	5.99
21	3.10	0.20	143.00	153.20	148.60	169.40	4.07	7.12
22	13.25	0.40	147.40	162.20	152.00	162.30	1.70	8.73
23	5.89	1.05	128.20	140.20	133.60	151.40	2.96	6.49
24	4.40	0.34	160.50	160.80	162.50	169.80	6.36	8.36
25	7.28	0.00	131.19	143.43	134.00	161.90	3.41	6.49
26	9.00	0.60	138.40	153.10	116.70	157.80	2.67	9.37
27	3.94	0.00	138.90	147.40	136.50	154.70	3.24	5.82
28	9.16	0.10	122.40	147.20	134.20	150.90	2.02	8.56
29	8.04	0.79	114.70	129.40	123.20	147.70	3.16	7.83
30	10.72	2.43	129.20	143.40	130.40	153.20	2.06	4.34
31	9.50	0.62	131.90	149.10	140.70	159.90	2.73	5.79
32	13.14	0.78	123.00	135.50	138.50	154.60	3.80	11.49
33	4.67	1.58	104.20	140.70	149.10	158.60	4.33	8.83
34	3.89	0.63	124.80	138.50	145.60	148.60	2.52	7.54
35	8.09	1.94	128.20	142.60	117.70	147.10	3.95	14.10
36	11.30	1.20	134.20	151.20	85.40	156.80	3.40	7.38
37	17.23	0.30	108.40	145.40	103.40	141.60	2.87	14.43

*ADI* atlantodental interval, *CCA* clivus-canal angle, *CMA* cervicomedullary angle, *SLMI* sum of lateral mass interspace

### Surgical procedure

All patients received posterior reduction, screw fixation, bone grafting, and fusion. Selective transoral or retropharyngeal release was performed before the posterior procedure depending on the reduction rate under skull traction [14, 15]. Patients with atlas occipitalization underwent occipital-cervical fusion, and patients not with that underwent atlantoaxial fixation and fusion except one patient who underwent occipital-cervical fixation because of failure in atlas lateral mass screw insertion. All of the patients underwent posterior reduction and internal fixation by pedicle screw and rod: using C1 lateral mass screw and C2 pedicle screw fixation in 19 patients, and using occipitocervical fixation in 18 patients (9 patients for C0–3, 8 patients for C0–2 and 1 patient for C0–4). Transoral release was completed in 2 patients, and retropharyngeal release in 4 patients (Table 1).

The Gardner Well skull traction with 1/6 body weight was applied under general anesthesia. Transoral or retropharyngeal release was performed if reduction was not satisfactory under skull traction. The details of transoral and retropharyngeal release were reported in the article by C Wang et al. [16] and Hao et al. [17]. Posterior fixation was performed with a pair of pedicle screws (DePuy Synthes Companies, Raynham, MA) in the axis, a pair of lateral mass screws (DePuy Synthes Companies, Raynham, MA) in the atlas (for atlantoaxial fusion) according to Goel et al. [18], and a titanium plate (DePuy Synthes Companies, Raynham, MA) in the occiput (for occipital-cervical fusion). Posterior arch of the atlas, lamina, or axis, and the occiput (only when the occiput was fixed) were decorticated, and fused by morselized cancellous bone harvested from the posterior-superior iliac crest. Cervical X-ray, CT and MRI were obtained 3~5 days after the procedure for further measurements. Cervical X-ray and/or CT were obtained at the 3 months, 6 months and 1 year follow-up to evaluate the status of fusion and instrumentation.

### Radiological measurements

**Fig. 1:**
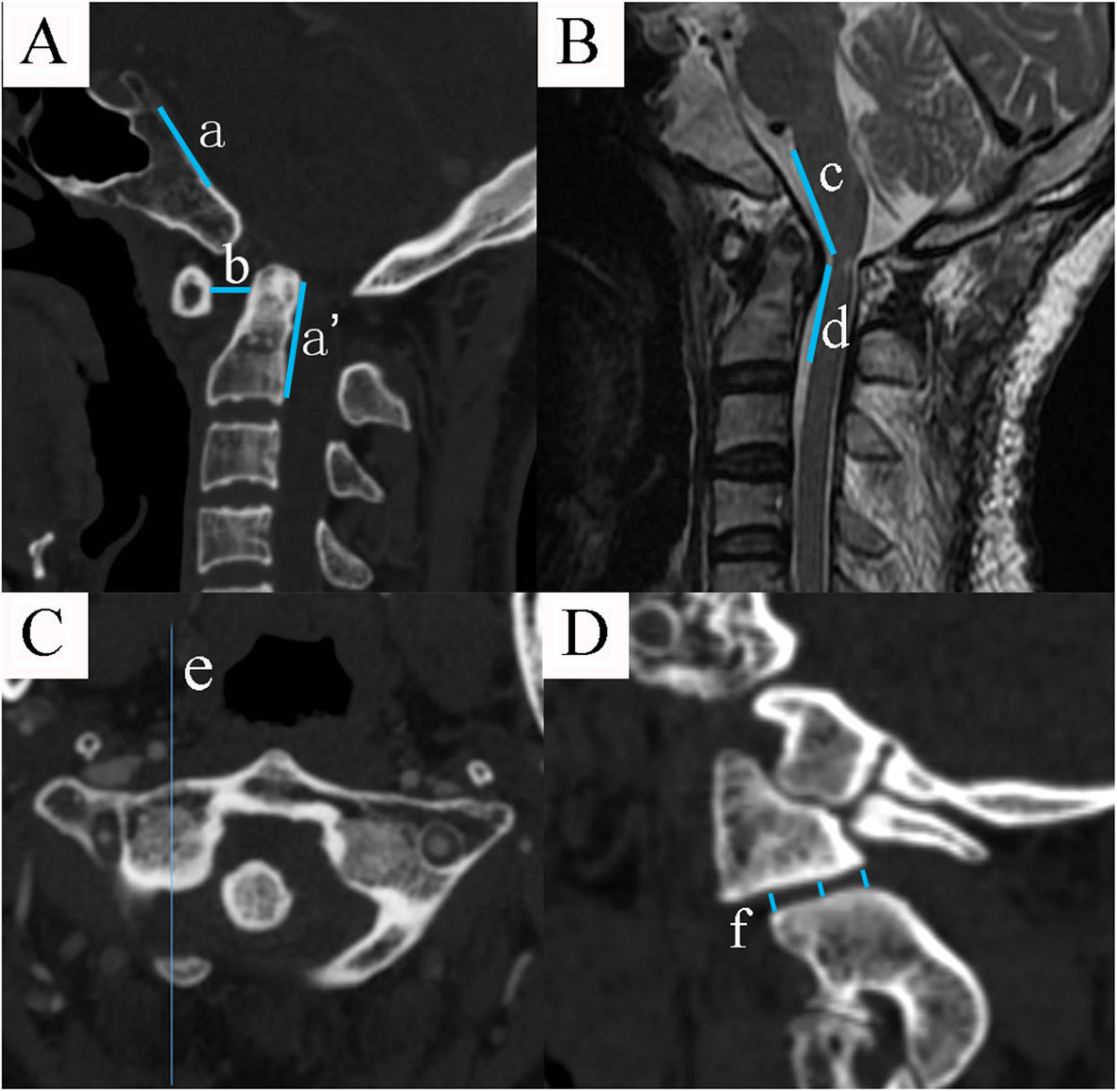
**a** The crossing angle of the line of inferior 1/3 clivus (line a) and the line extending from the posterior border of the dens to the posterior-inferior border of the axis body (line a’) was defined as clivus-canal angle (CCA). The distance from the posterior border of the anterior C1 arch to the anterior border of the odontoid process (line b) was measured as anterior atlantodental interval (ADI). **b** The crossing angle of the line extending the anterior border of the ventral medulla (line c) and the line extending the anterior border of the ventral upper cervical spinal cord (line d) in midline sagittal T2 MRI was defined as cervicomedullary angle (CMA). **c** and **d** The middle line of the lateral mass in the cross-sectional slice was defined as line e. The corresponding sagittal reconstruction CT based on line e was seen in D. We measured the anterior part, middle part, and posterior part of the lateral mass interspace, and the average value was defined as the lateral mass interspace (LMI). The combined bilateral LMI was defined as sum of LMI (SLMI)

We measured pre-operative and post-operative (3~5 days after the procedure) ADI, CCA, CMA, sum of lateral mass interspace (SLMI) of the operation and the control group. Measurements of ADI and CCA were based on the midline sagittal reconstruction CT image. Carestream software (Carestream Health Inc., Rochester, NY) was used to perform the measurement. ADI, defined as the distance from the posterior border of the anterior C1 arch to the anterior border of the odontoid process, was measured. CCA, defined as the angle formed between the line of inferior 1/3 clivus and the line extending from the posterior border of the dens to the posterior-inferior border of the axis body, was measured. CMA, the angle formed between the line extending the anterior border of the ventral medulla and the line extending the anterior border of the ventral upper cervical spinal cord, was measured using the midline sagittal T2 MRI. Lateral mass interspace (LMI) was measured using the sagittal reconstruction CT based on the cross-sectional slice corresponding to the middle line of the lateral mass. We measured the anterior part, middle part, and posterior part of the lateral mass interspace, and the average value was defined as the LMI (Fig. 1). The combined bilateral LMI was defined as SLMI. All parameters were measured thrice, and the average value was adopted for further analysis.

### Statistical analysis

SPSS 20.0 for Windows PC version (SPSS Inc., Chicago, IL) was used for statistical analysis. Independent *t* test was conducted to compare the age between the operation group and the control group, and χ^2^ test was conducted to compare the sex between the two groups. One-way analysis of variation (ANOVA) was conducted to compare ADI, CCA, CMA, and SLMI between the pre-operative and post-operative conditions of the operation group and the control group. *P* < 0.05 was considered as statistical difference.

## Results

The average age in the operation group and the control group were 47.8 ± 12.1 years and 49.8 ± 10.3 years, respectively. Independent *t* test showed no statistical difference of age between the operation group and the control group (*t* = 1.98, *p* > 0.05), and χ^2^ test showed no difference of sex between the two groups (χ^2^ = 0.402, p > 0.05).

Instrument failure or loss of reduction were observed in 3 patients after occipital-cervical fixation. One patient encountered unilateral rod breakage 3 months after the procedure, and two patients presented with loss of reduction 6 months after the procedure even though the consecutive sagittal CT scan indicated bony fusion.

**Fig. 2:**
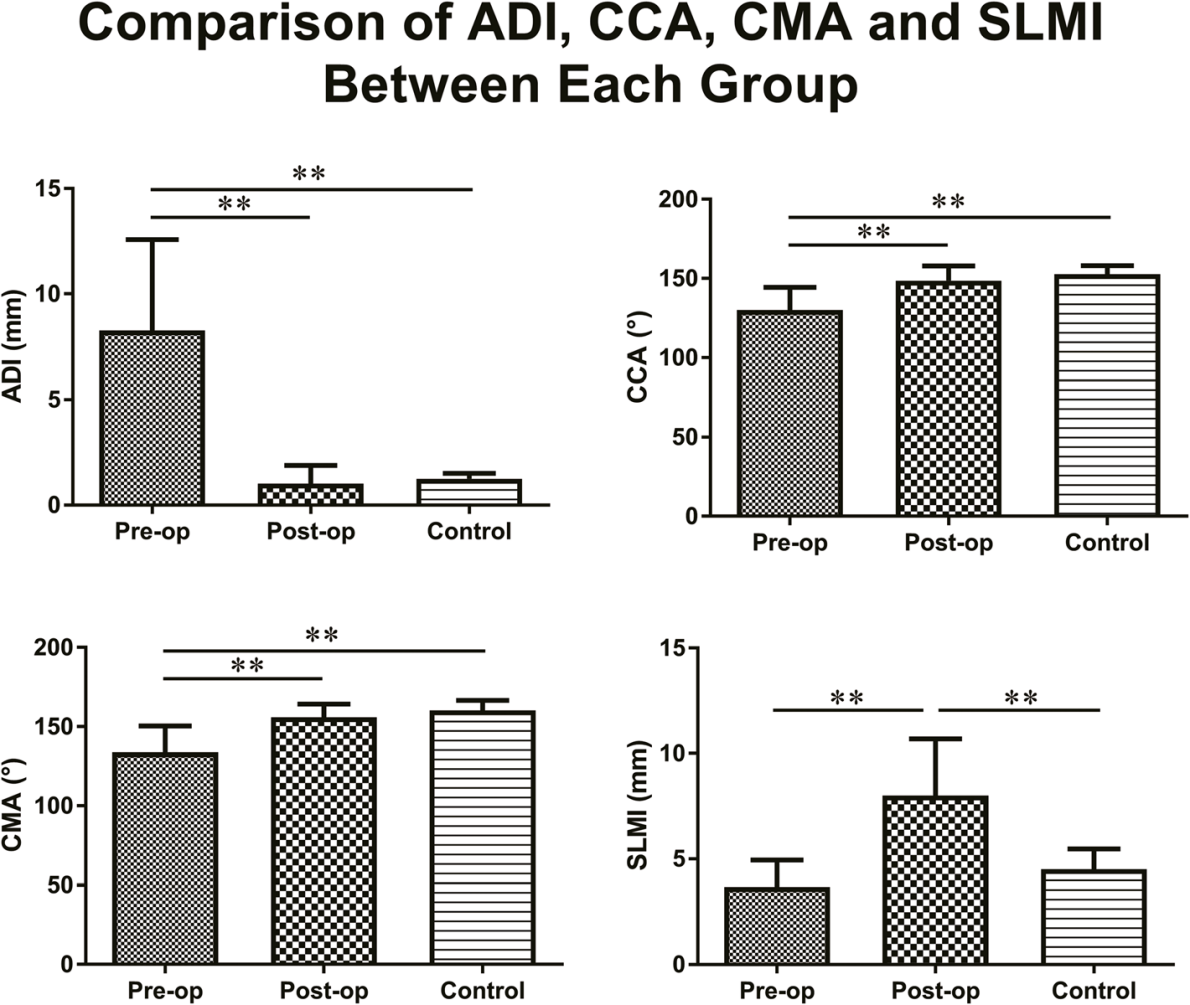
Post-operative atlantodental interval (ADI), clivus-canal angle (CCA), cervicomedullary angle (CMA), and sum of lateral mass interspace (SLMI) were statistically different from pre-operative assessments. Pre-operative ADI, CCA, and CMA values were statistically different from those of the control group, while the SLMI has no significant difference between the pre-operative condition and the control group. Post-operative ADI, CCA, and CMA had no significant difference compared to those of the control group, while post-operative SLMI was statistically different from that of the control group. ** indicates *p* < 0.01

The ADI, CCA, CMA, and SLMI in the pre-operative condition of the operation group were 8.3 ± 4.3 mm, 130.2 ± 14.2°, 133.8 ± 16.7°, and 3.7 ± 1.3 mm, respectively, those in the post-operative condition of the operation group were 1.0 ± 0.9 mm, 148.5 ± 9.4°, 156.0 ± 8.2°, and 8.0 ± 2.7 mm, respectively, while those in the control group were 1.2 ± 0.3 mm, 152.7 ± 5.3°, 160.2 ± 6.3°, and 4.5 ± 1.0 mm respectively. Post-operative ADI, CCA, CMA, and SLMI were statistically different (*p* < 0.01) from pre-operative assessments with F values being 85.8, 43.7, 50.9, and 54.5, respectively (for more details in Table 2). Pre-operative ADI, CCA, and CMA values were statistically different from those of the control group, while the SLMI has no significant difference between the pre-operative condition and the control group. Post-operative ADI, CCA, and CMA had no significant difference compared to those of the control group, while post-operative SLMI was statistically different from that of the control group (Fig. 2).

## Discussion

Surgical procedures for treating atlantoaxial dislocation include odontoid resection, laminectomy and decompression of foramen magnum, posterior reduction and internal fixation [19–21] or combined anterior release, and posterior reduction and fixation [2, 22, 23]. Galli and Brooks together pioneered posterior fixation using stainless steel wire. Appofix was as an alternative to fixation at one time. These techniques achieve fixation of only the posterior column. Thus, they have less biomechanical strength [22]. Transarticular fixation technique (Magerl, et al) [24], plate and screw fixation (Goel, et al) [18] and polyaxial pedicle screw technique (Harms, et al) [25] achieve fixation for three columns, and have been widely used for the past few years. Although with excellent biomechanical strength, the Magerl technique was only suitable for fixation under correct anatomical position and alignment before the fixation [26]. The Goel-Harms technique, with its reductive property, has been more frequently used. However, we have observed instrumentation failure and loss of reduction for this technique. All the 3 failed patients underwent the occipital-cervical fixation, and no instrumentation failure was observed in C1–2 fixation. We suspect that because of the long arm of force of the occipital-cervical fixation, combining with the widening of the lateral atlantoaxial joints, instrumentation suffers hyper physiological loading and is liable to failure leading to the instrument breakage or loss of reduction. The C1–2 fixation system is speculated to be more reliable than the occipital-cervical system based on indirect biomechanical comparison [27, 28], unfortunately, no direct biomechanical comparison between the C1–2 fixation system and the occipital-cervical system has been reported.

**Fig. 3:**
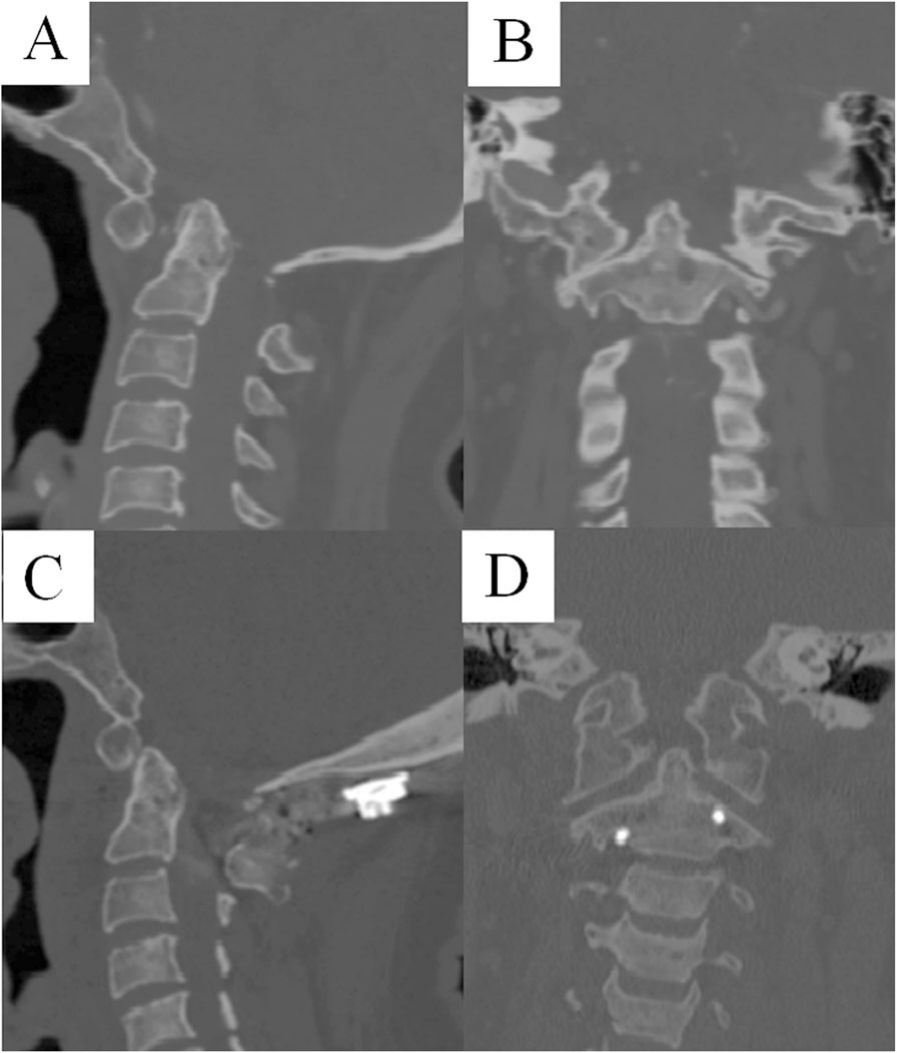
Case 26, a 57 years old female diagnosed as atlantoaxial dislocation and underwent retropharyngeal release and posterior C0–2 fixation. Pre-operative CT scan indicated atlantoaxial dislocation (**a**) and lateral atlantoaxial joints (**b**) in coronal reconstruction. Post-operative CT indicated anatomical reduction (**c**) and widening of lateral atlantoaxial joints (**d**)

ADI, CCA, and CMA are frequently used to evaluate the reductive effect after surgical treatment. Our data indicated anatomical reduction according to the post-operative ADI, CCA and CMA. We also found that CMA has approximately 7.5° more than CCA, so we recommend considering both CCA and CMA to more reliably evaluate the reduction. Post-operative SLMI was significantly higher than the pre-operative and control group (Figs. 2, 3). Despite the LMI being greater than in normal condition, the instrumentation may deform to release stress. The screw and plate system created by C Wang et al. had no sliding hole in the early stage and had a high rate of instrumentation failure. When the hole in the plate was changed to sliding type, the rate of instrumentation failure significantly decreased.

Some scholars attempted lateral mass bone grafting or cage implantation to assist the fusion of the atlantoaxial joint [19, 29]. This type of bone grafting is similar to the interbody fusion techniques used in the lumbar spine, and has a high fusion rate. Our results indicate that because of the widening of the lateral mass joint, bone grafting or cage implantation of the lateral mass could reduce the load of instrumentation and may decrease the rate of instrumentation failure. However, the SLMI was only 8.0 mm in post-operative condition based on our study, thus, mean of unilateral lateral mass interval is only about 4.0 mm in post-operative condition. Additionally, the lateral mass joint has an irregular shape and there is no individualized cage for lateral mass implantation, therefore, it is not suitable for cage implantation. After a cage was implanted to the lateral mass, it may lead to a reverse opening of the lateral mass (anterior end closed and posterior end opened) if the cage migrates or was not implanted appropriately, and thus, adversely affecting the CCA and CMA. We recommend lateral mass bone grafting rather than cage implantation according to our experience because the former technique is much easier to perform and the cancellous morselized bone can adjust to the variable shape of the lateral mass joint.

We compared the radiographic parameters pre- and post- reductive procedure of the atlantoaxial dislocation via posterior fixation using pedicle screw and rod in this study. To the best of our knowledge, this is the first report on the lateral mass interspace. Our results indicate the widening of the lateral mass after reduction and fixation of the atlantoaxial dislocation using pedicle screw and rod, and this condition exists in both atlantoaxial fusion and occipital-cervical fusion. Widening of the lateral mass leads to excessive stress of hardware and loss of atlantoaxial reduction. The limitation of this study is its retrospective nature and the sample size which is not very large. We will compare the parameters between atlantoaxial fusion and occipital-cervical fusion in future studies with a larger sample size to provide adequate data for further validating the results of this study.

## Conclusions

The lateral mass joints were widened after the anatomically reduction of atlantoaxial dislocation by pedicle screw and rod.

Widening of the lateral mass exists in both atlantoaxial fusion and occipital-cervical fusion.

## Data Availability

The datasets used and/or analysed during the current study are available from the corresponding author on reasonable request.

## Abbreviations

ADI: Atlantodental interval
ANOVA: Analysis of variation
CCA: Clivus-canal angle
CMA: Cervicomedullary angle
CT: Computerized tomography
LMI: Lateral mass interspace
MRI: Magnetic resonance imaging
SLMI: Sum of lateral mass interspace
